# Ischemic Risk in Patients with Zero Coronary Artery Calcium Undergoing Stress PET/CT Testing

**DOI:** 10.3390/jcm15176603

**Published:** 2026-08-26

**Authors:** Jeffrey L. Anderson, Leslie K. Iverson, Daniel L. Bride, Stacey Knight, Raymond O. McCubrey, Viet T. Le, Mohanad Dakhakhni, Steve M. Mason, Kirk U. Knowlton

**Affiliations:** 1 Intermountain Medical Center Heart Institute, 5171 So. Cottonwood Street, Building 1, 5th Floor, Murray, UT 84107, USA; leslie.iverson@imail.org (L.K.I.); daniel.bride@imail.org (D.L.B.); stacey.knight@imail.org (S.K.);; 2 The University of Utah School of Medicine, 30 N 1900 E, Salt Lake City, UT 84132, USA; 3 Rocky Mountain University of Health Professions, 1800 S Novell Pl, Provo, UT 84606, USA

**Keywords:** coronary artery calcium, coronary artery disease, microvascular disease, prognosis, risk factors

## Abstract

**Background**: The absence of coronary artery calcium (CAC = 0) on computed tomography (CT) portends a low but not zero coronary risk. However, the frequency and prognostic significance of non-calcified plaques in CAC = 0 patients are incompletely defined, and non-coronary artery disease (CAD) causes of coronary risk have been proposed. We sought to define the ischemic burden, CAD status, and outcomes of CAC = 0 patients. **Methods**: The Intermountain electronic medical records were searched for CAC = 0 patients undergoing stress positron emission tomography (PET)/CT between January 2016 and July 2022. We defined definite PET abnormality as ischemic burden (IB) ≥ 10%. Patients were followed for angiographic findings, interventions, and major adverse cardiovascular events (MACE) over 3.1 ± 1.3 years. **Results**: Of 22,542 PET/CT studies in primary prevention patients, 5054 (22.4%) had CAC = 0. Of these, 59 (1.2%) had IB ≥ 10% on PET. Selective coronary angiography was performed within 90 days in 25 (42%) of IB ≥ 10% patients. Severe CAD was present in 11, but findings were not explained by CAD in 14. Revascularization was indicated in 10 (16.9%) of 59 with IB ≥ 10% vs. 0.04% of IB = 0% (*p* < 0.0001); 3-year outcomes also were lower with IB = 0%. **Conclusions**: In this large series of stress PET/CT study data, IB ≥ 10% in patients with CAC = 0 was rare. Most positive PET tests were not explained by obstructive CAD. While CAC does not perfectly predict an absence of coronary ischemia or obstructive CAD, it is a strong marker of prognosis. Here, we show that IB by PET supplements CAC score to importantly refine risk assessment.

## 1. Introduction

Coronary artery calcium (CAC) on computed tomography (CT) has been shown to be a strong and incremental risk factor for coronary events [1,2,3,4,5,6]. In contrast, the absence of coronary artery calcium (CAC = 0) on CT portends a low but not zero coronary risk [3,7,8]. However, the frequency and prognostic significance of non-calcified plaques in CAC = 0 patients are poorly defined. Also, the contribution to coronary events of alternative pathologies in CAC = 0 patients, such as spontaneous coronary artery dissection (SCAD), coronary spasm, microvascular disease, coronary thromboembolism, coronary anomalies, etc., is incompletely understood [9]. This is especially true for symptomatic populations undergoing combined cardiac stress positron emission tomography (PET) and computed tomography (CT) testing (PET/CT).

Intermountain Health has long and extensive experience with PET/CT testing in patients at primary risk for coronary artery disease (CAD) [10,11]. We examined Intermountain Health’s nuclear medicine and electronic medical records (eMR) databases to determine the prevalence and ischemic risk burden of patients who were referred for PET/CT, who were at primary CAD risk, and in whom the CT study indicated a zero CAC score.

## 2. Methods

### 2.1. Study Design and Objectives

This was a retrospective, observational study. The study objectives were as follows: (1) to define the prevalence of CAC = 0 patients among all those at primary CAD risk referred for a PET/CT study for an elevated coronary risk or symptoms of a potentially cardiovascular (CV) nature or the need for definitive, pre-intervention risk assessment (e.g., before non-coronary CV surgery, renal transplantation, or antiarrhythmic drug initiation); (2) among those with CAC = 0, to categorize the distribution of ischemic burden (IB) by PET; (3) among those referred for coronary angiography after PET/CT (typically for positive IB on PET and clinical concern for CAD), to summarize the angiographic findings and subsequent coronary interventions; and (4) to determine the 90-day and 3-year CV outcomes (i.e., incidence of major adverse cardiovascular events [MACE]) in CAC = 0 patients.

### 2.2. Study Population, Definitions, and IRB Approval

To identify the study population, the Intermountain Health’s nuclear medicine and hospital eMR databases at 4 hospitals were searched for CAC = 0 patients undergoing PET/CT between 1 January 2016 and 1 July 2022 and who were at primary atherosclerotic cardiovascular disease risk (i.e., patients with no prior history of myocardial infarction [MI], stroke, or coronary intervention). If symptomatic (e.g., chest pain, dyspnea, or other), symptoms were variable and atypical enough to favor PET/CT over a direct referral for coronary angiography to define coronary risk but, together with other risk features, sufficient to recommend the more complex and expensive PET/CT procedure over CT testing alone. The most common diagnostic code used in our system to qualify PET/CT studies for insurance coverage is ICD-10 I20.89, which suggests concern for angina equivalent. A small percentage of patients (0.59%, *n* = 30) presenting with acute coronary syndrome (ACS) at the time of their PET/CT scan were omitted from the analysis as not satisfying the definition of primary prevention patients at the time of CAC = 0 determination.

We defined PET results for IB ≥10% as definitely positive, IB > 0 to <10% as equivocal, and IB = 0 as normal. The selection of IB ≥10% as definitely positive was based on major studies demonstrating that a cutoff of 10% LV ischemic burden differentiates those patients who would have lower risk of cardiac death after revascularization (≥10%) from those patients who would benefit more from medical therapy alone (<10%) [12]. We defined severe angiographic CAD as ≥70% diameter stenosis of a major coronary vessel, moderate as 50–69% stenosis, and mild as <50% stenosis.

Patients were followed in the eMR for coronary angiographic studies, coronary interventions, and MACE, including death, non-fatal MI, or revascularization before and after 90 days from PET/CT to the end of follow-up (at 3.1 ± 1.3 years).

As a database-only, observational study without study-related interventions, this study was approved by the Intermountain Health Institutional Review Board (IRB) with a waiver of consent.

### 2.3. PET/CT and CAC Protocols

The technical features of our PET/CT studies have been previously published [10,11]. Briefly, perfusion studies and CAC assessments were performed with a Siemens Biograph-16 True Point System-integrated 16-slice PET/CT scanner using the manufacturer’s modifications of the protocol previously described by Di Carli, et al. [13,14], utilizing rubidium-82 (82Rb) as the radionuclide emission agent. Following initial rest perfusion imaging, CAC was assessed by a non-enhanced 16-slice CT scanner triggered by prospective ECG gating, which generated 3 mm thick image slices during breath-holding. CAC scores were calculated by the Agatston method [15]. Stress perfusion imaging was then performed using regadenoson as the pharmacologic stressor. Perfusion and CAC results were taken from the clinical readings of nuclear board-certified or qualified cardiologists who were unaware of the study aims or results.

### 2.4. Decision to Perform Invasive Coronary Angiography (ICA)

In this retrospective observational study, the choice to perform ICA was independently made by the practicing cardiologist. Factors influencing the decision to proceed with ICA included not only the PET result but also the CAC score, other laboratory information, and, importantly, details of the clinical history (e.g., standard risk factor burden, associated clinical symptoms, whether typical or atypical for ischemia, and clinical suspicion of CAD), as well as shared decision-making discussions with the patient.

### 2.5. Study Outcomes

Consistent with the study objectives, we assessed the percentage of total PET/CT studies with CAC = 0. Of these, we defined categories of IB = 0, IB > 0 to <10%, and IB ≥ 10% based on results of the PET study. To eliminate artifactual abnormalities, the few nominally positive (≥10% IB) studies (i.e., severe motion artifact, diaphragmatic attenuation, etc.) were excluded (*n* = 7).

We next identified the patient subsets that underwent coronary angiography and summarized the coronary angiographic findings overall and by IB. We then assessed the proportion and type of coronary interventions performed (i.e., percutaneous coronary intervention [PCI] with stenting or coronary artery bypass graft surgery [CABG]). Finally, we evaluated patient outcomes in the peri-angiographic period and during longer-term follow-up. Outcomes of interest included coronary interventions and MACE, including all-cause death, non-fatal MI, or revascularization within and after 90 days from the time of the PET/CT study through 3.1 ± 1.3 years of follow-up.

### 2.6. Statistical Considerations

Variables of interest are summarized as means +/- SD or as medians and interquartile range as appropriate. To examine differences among the various categories of IB or of CAC scores, Fisher’s exact test (for categorical variables) and analysis of variance (for continuous variables) tests were used. We also performed a post-hoc power analysis to determine the smallest relative risk increase we could have detected for total MACE in those with IB > 10% vs. IB = 0%. Given our observed total MACE in those with IB = 0% of 4.5% (see Section 3), we were able to detect a relative risk increase of 1.75 or an increase in the absolute MACE rate to 7.9% with a power = 0.90 and alpha = 0.05.

## 3. Results

### Baseline Characteristics and IB Findings

The search of Intermountain Health’s nuclear medicine and eMR database identified 52,760 total PET/CT studies over the period from 1 January 2016 to 1 July 2022. Of these, 22,542 were for primary atherosclerotic cardiovascular disease risk assessment, of which 5084 (22.6%) had a CAC score of zero. Of these, 5054 tested patients had no ACS on presentation for PET/CT. IB distribution on PET included 4479 (88.6%) with IB = 0, 516 (10.2%) with IB > 0 to <10%, and 59 of adequate quality (1.2%) with IB ≥ 10%. This indicates a negative predictive value (NPV) of 98.8% for IB ≥ 10% with CAC = 0. In contrast, 54% of the overall PET/CT database had IB ≥ 10%.

Baseline characteristics by IB are shown in Table 1 for the CAC = 0 primary prevention cohort, and they were generally similar across IB categories with a few exceptions. BMI was lower in the IB = 0 group. Also, the percentage with a diagnosis of hyperlipidemia increased across increasing IB categories. Statin use was low at baseline (10–22%). The course of these primary prevention patients undergoing PET/CT is shown in the Graphical Abstract.

Invasive coronary angiography (ICA), interventions, and outcomes across IB categories are reported in the Graphical Abstract (Table 2). ICA was clinically indicated within 90 days, based on all clinical and laboratory findings, in 25 (42.4%) of the 59 CAC zero patients with IB ≥ 10% and 95 of 4995 (1.9%; *p* < 0.0001) of IB,10% elective cohort patients (Table 2).

Overall, a CAC score of zero was associated with low outcome rates and high NPV values, including for IB ≥10% (59/5054 = 1.2%, NPV 98.8%), severe (≥70% diameter stenosis) CAD (19/5054 = 0.4%, NPV 99.6%), revascularization in <90 days (15/5054 = 0.3%, NPV 99.7%), MACE in <90 days (33/5054 = 0.7%, NPV 99.3%), and total MACE (247/5054 = 4.9%, NPV 95.1%).

There was an interaction between zero CAC and IB. For IB 0–<10%, event rates were even lower and NPV values higher, i.e., severe CAD (8/4995 = 0.2%, NPV 99.8%), revascularization in <90 days (5/4995 = 0.1%, NPV 99.9%), MACE in <90 days (23/4995 = 0.5%, NPV 99.5%), and total MACE (231/4995 = 4.6%, NPV 95.4%).

In contrast, event rates were higher and NPV values substantially lower for zero CAC with IB ≥ 10%, i.e., severe CAD (11/59 = 18.6%, NPV 81.4%), revascularization or MACE in <90 days (10/59 = 16.9%, NPV 83.1%), and total MACE (16/59 = 27.1%, NPV 72.9%).

Severe CAD (defined as ≥70% diameter stenosis) was present in 11 (18.6%), and revascularization was required in 10 (16.9%) (5 with CABG only, 4 with PCI only, and 1 with both PCI and CABG). Two of those with severe CAD were thought to have a congenital stenosis of the ostial left circumflex coronary artery rather than classical atherosclerosis.

Thus, 14 (56%) of the 25 angiographic cases and 48 (81.4%) of the 59 patients with IB ≥ 10% were not explained by severe epicardial CAD. No cases of SCAD were noted in our series. Findings in those patients with a positive PET test but without epicardial obstructive CAD suggest a microvascular etiology for their perfusion abnormalities.

ICA was performed for clinical indications in far fewer patients with equivocal IB (IB >0–<10%), i.e., 56/516 or 10.9%, with only four found to have severe CAD (0.8%) and only three (0.6%) requiring revascularization.

In contrast, ICA was undertaken in only 39 (0.9%) of the 4479 IB = 0 elective patients. Severe CAD was found in only 4 of the 4479 with IB = 0 (0.1%), and early revascularization was indicated in only 2 (0.04%).

**Figure 1:**
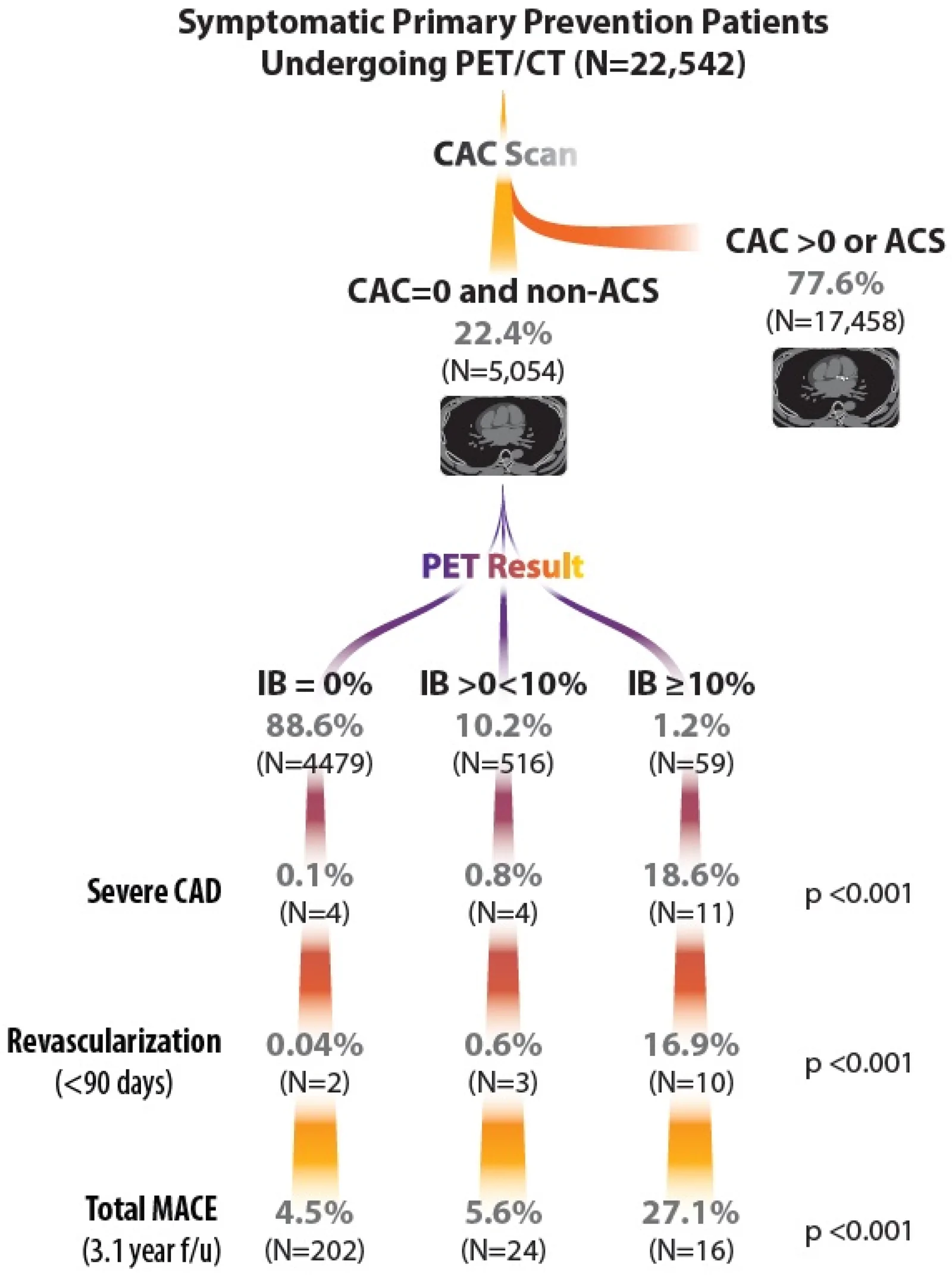
Graphical Abstract of Study Flow.

Differences across IB categories for coronary angiography, findings of obstructive CAD, and coronary interventions were highly significant (*p* < 0.0001) (Figure 1, Table 2). MACE within 90 days occurred in 10 (16.9%) of 59 with IB ≥ 10% (all revascularizations) vs. 0.6% of IB > 0 to <10% and 0.04% of IB = 0% (*p* < 0.0001). Rates of MACE to the end of follow-up (averaging 3.1 years) occurred in 27.1% with IB ≥ 10%, 5.6% of IB > 0 to <10% and 4.5% of IB = 0% (*p* < 0.0001) (Figure 1, Table 2).

Components of MACE are shown in Table 2. The excess of events at ≤90 days was driven by revascularizations in the IB ≥ 10% group. Events with IB ≥ 10% at >90 days trended to be higher for death and revascularization. Overall, the excess of MACEs with IB ≥ 10% was driven by revascularization, with a numerical increase in deaths.

## 4. Discussion

### 4.1. Summary of Study Findings

In this large single healthcare system clinical database, we found the prevalence of CAC = 0 among patients referred for PET/CT because of concern for elevated coronary risk or symptoms of a potentially coronary etiology to be 22.6%, which is similar to or lower than in previous reports (see Section 4.2, below) and indicates ours to be a relatively high-risk patient referral cohort. However, even among this higher-risk referral population CAC = 0, IB ≥ 10% was rare (1.2%). The frequency of coronary procedures correlated with IB, but overall, in CAC = 0 patients, coronary angiography (2.49%) and coronary revascularization (0.23%) were rare. Examination of coronary angiographic findings in these patients revealed that severe, obstructive CAD explained a positive PET scan in only 11 (18.6%) of 59 patients with IB ≥ 10% PET studies, but 14 (56%) of angiographic patients and 48 overall of these 59 (81%) did not have obstructive CAD. Coronary stenosis was ascribed to a congenital coronary anomaly in two cases; others appeared to be non-calcified obstructive plaques. There were no cases of SCAD or other epicardial coronary pathology in our series. Thus, the majority of PET-positive, CAC = 0 cases did not have an obstructive CAD or other epicardial coronary artery explanations, which suggests a microvascular etiology for their perfusion abnormalities. The 4.8% event rate at a mean of 3.1 years is low but not negligible, indicating the need to integrate CAC results with PET and other clinical findings.

### 4.2. Literature Comparisons

The present study represents the largest single healthcare system experience with zero CAC in patients without a history of CAD undergoing cardiac PET/CT studies. This 5054 CAC = 0 patient experience defines the IB distribution and MACE outcomes and importantly adds to previous reports, including in CAC = 0 patients evaluated by CT coronary angiography (CTCA).

Patients referred for PET for ischemia plus CT for CAC determination represent a higher-risk population than those referred for stand-alone CT for CAC. This includes a higher clinical suspicion of ischemia.

PET offers more diagnostic accuracy than single-photon emission computed tomography in testing for inducible myocardial ischemia, although studies with both imaging techniques have observed zero to low CAC scores to be infrequently associated with ischemia, whereas there is a wide variance in the frequency of ischemia among patients with CAC > 0 scores [16].

Relatively small earlier studies with PET/CT provided limited data but suggested that zero CAC predicts a very low risk of inducible ischemia [17,18]. In previous research, Rozanski et al. (2023) reviewed 3307 patients referred for PET/CT, of whom 237 (25%) had CAC = 0 [19]. This finding was associated with a low frequency of IB ≥ 5% (, i.e., ≤1.5% across ages). Frey et al. (2023) analyzed a 2640-patient PET/CT database and proposed that zero CAC could be used to avoid the cost and radiation of perfusion studies [20]. Zero CAC was present in 26%, of which only 2.6% had IB ≥ 10% (NPV 97%) vs. 27.6% with CAC present.

In contrast, Patel et al. argued that use of anatomic testing such as CAC = 0 to avoid myocardial perfusion imaging in symptomatic patients could lead to missed diagnosis of microvascular dysfunction (as assessed by myocardial blood flow reserve), which was a risk contributor in their database of 5983 primary risk patients undergoing PET/CT, of whom 22% had CAC = 0 [21]. We similarly found a high percentage of PET/CT positive CAC = 0 patients without obstructive CAD in whom microvascular dysfunction was suggested.

CAC = 0 also has been evaluated as a predictor of obstructive CAD in various series of patients undergoing CTCA. CTCA has an advantage over perfusion studies in that it directly assesses coronary anatomy and plaque morphology, including non-calcified plaques. However, it has the disadvantage of being less accurate in determining the prognostic and therapeutic implications of coronary disease compared with assessments of coronary perfusion or flow (e.g., as with fractional flow reserve [FFR] measurements) [22]. In the 10,037-patient CONFIRM Registry, only 3.5% of CAC = 0 patients had ≥50% stenoses [23]. NPV for CAC = 0 and ≥50% stenosis was 96%. Of those with stenoses, MACE risk was low although moderately greater with than without stenoses (3.9% vs. 0.8% over 2.1 y), but mortality did not differ.

Mittal et al. reported on 2730 patients undergoing CTCA with stable symptoms, of whom 52% had CAC = 0 [24]. Moderate stenoses (50–69%) were found in 1.2% and severe stenoses (≥70%) in 0.5%, which yielded an NPV of 99.5% for excluding ≥70% coronary stenoses. Annual event rates were lower with CAC = 0 than CAC ≥ 1 (0.3% vs. 1.2%). Notably, the presence of non-calcified atheroma did not affect prognosis. They proposed that a zero CAC score could be considered as a ‘gatekeeper’, obviating further, expensive diagnostic testing.

Wang et al. [8] noted that only 1.9% of 915 patients undergoing CTCA with CAC = 0 had a ≥50% stenosis. A total of 915 (52%) of 1753 patients had zero CAC scores. On CTCA, 8.4% had non-calcified CAD with <50% stenosis and 1.9% had ≥50% stenosis (NPV for ≥50% stenosis, 98.1%). Annualized MACE rates over 2.2 years were 0.6% and 2% for zero- and non-zero CAC, respectively. They concluded that zero CAC in patients undergoing CCTA has a high NPV for excluding non-calcified obstructive CAD and was associated with excellent medium-term prognosis.

Agha et al. presented a meta-analysis of 32 studies involving 92,279 patients, addressing the prognostic value of zero CAC among patients with either stable or acute chest pain and undergoing CTCA [25]. CAC = 0 was reported in 45% with stable and 58% with acute chest pain. The NPV for ≥50% coronary stenoses was 97%, and it was 98% for stable and acute chest pain, respectively. Zero CAC predicted a low annualized MACE rate with both stable (0.5% with CAC = 0 vs. 2.2% with CAC ≥ 1) and acute chest pain (0.8% for CAC = 0 vs. 8.5% for CAC ≥ 1). They concluded that zero CAC in both chest pain presentations was associated with a low prevalence of non-calcified CAD and a low annualized risk of MACE. They proposed that CAC = 0, in a value-based health care delivery system, could serve as a ‘gatekeeper’ for more advanced imaging studies in patients presenting with chest pain.

CAC has also been reported to be of incremental value with regard to the recent era of COVID-19. In the Italian (S) Core COVID-19 Registry, the coronary calcium score was found to contribute to stratifying the risk of complications in hypertensive COVID-19 patients [26].

### 4.3. Mechanistic Considerations

The low risk noted in zero-CAC subjects has been ascribed to non-calcified, “soft” plaques. However, the frequency and prognostic significance of non-calcified plaques in CAC = 0 patients are poorly defined, as noted in the previous literature reports. These combined results argue for a high NPV of CAC = 0 (>98%). Also, the contributions to coronary events of alternative pathologies in CAC = 0 patients, such as SCAD, coronary spasm, microvascular disease, coronary thromboembolism, coronary anomalies, etc., are incompletely understood [9]. This study addresses the distribution of coronary pathologies in CAC = 0 patients with a positive PET test who then underwent angiographic assessment. The findings indicate that IB ≥ 10% is rare. In these patients, a variable degree of non-calcified coronary plaques was often present; however, nonobstructive disease or no angiographic disease was found to be even more frequent than obstructive disease. Indeed, angina with nonobstructive coronary artery disease (ANOCA) is being increasingly recognized and may comprise half or more of cases of suspected coronary ischemia undergoing angiography [27,28]. The leading candidate mechanism to explain ANOCA is microvascular dysfunction. We have observed that microvascular dysfunction also underlies the vascular pathology of acute stress cardiomyopathy [29].

Recently, artificial intelligence-based quantitative analysis of CCTA images has been reported for patients with chest pain and zero CAC by clinical readings. Non-calcified plaques predominated, but small calcifications were not uncommon. Despite this, long-term clinical outcomes were generally good [30].

### 4.4. Clinical Implications

This study, which represents the largest single cohort of cardiac PET/CT patients, confirms the excellent medium-term prognosis reported in association with zero CAC. It also provides a thorough analysis of the diverse types of coronary pathology encountered in these patients. However, we believe that the use of CAC = 0 as a ‘gatekeeper’ in considering whether more advanced imaging studies should be pursued with caution. For example, Smettei et al., studied 1888 symptomatic adults with coronary risk factors who had a CAC score of zero. Of these, 2.2% had a coronary stenosis of ≥70%. These patients were younger (average age 45 vs. 59 years). They concluded that a zero CAC does not rule out significant CAD in young adults with chest pain and with CAD risk factors. These patients may need further investigation [31]. Here, we show the complementary nature of CAC and IB by PET in this population. Given the gradient of risk of obstructive CAD in the IB ≥ 10% category, we believe that ICA can be recommended in the appropriate clinical context, i.e., when taken together with patient symptoms and risk factors and the clinical need for precise coronary diagnosis in the IB ≥ 10% subgroup. In summary, given the occurrence, although rare, of true high-grade obstructive, non-calcified coronary plaques, zero CAC should not be used in isolation for decision-making but in association with the full context of clinical information when deciding whether to proceed with more advanced coronary assessments such as by PET, CTCA, or ICA.

### 4.5. Study Strengths and Limitations

This study has the strength of a large and consecutive population of patients undergoing referral for cardiac PET/CT for symptoms or for precision risk assessment in a single model US healthcare system with a consistent and homogeneous approach to patient referral and study performance and analysis.

Important limitations include a selected PET/CT referral population, possible angiographic verification bias, a MACE endpoint largely driven by revascularization events, and an indirect microvascular interpretation that requires further validation. The small number of CAC = 0 patients with IB ≥ 10% limits statistical power and mechanistic inferences in this subset.

As with all retrospective observational studies, it runs the risk of selection biases, some of which may be unknown or unrecognized and uncorrected for in comparative analyses. Our population is predominantly non-Hispanic White of Northern European heritage; application to other racial or ethnic groups may differ. MACE evaluation was based on Intermountain Health’s eMR records; a small percentage of events occurring outside of Intermountain Health may not have been captured in our eMR. Our database does not consistently and reliably separate coronary deaths from non-coronary and other cardiac or non-cardiac deaths, so total deaths are reported, which is less specific to a coronary-risk and -outcomes assessment. While we propose that microvascular dysfunction may be responsible for ischemic responses in many non-obstructive cases, the number of IB ≥ 10% patients with ischemia by PET is small, with limited power to compare the proportion of occlusive versus non-occlusive CAD cases. Further, no myocardial blood flow reserve data were consistently available in our database to demonstrate this. Finally, the 3-year average follow-up time limits conclusions about longer-term associations with MACE outcomes, so that longer-term follow-up is indicated.

## 5. Conclusions

In this large cohort study of cardiac stress PET/CT, findings of IB ≥10% in patients with CAC = 0 were rare (1.2%), as was obstructive CAD (0.4%). In these patients, PET was complementary to CT/CAC in identifying an enriched population with the presence of obstructive epicardial disease or microvascular angina and guiding further assessment and treatment. These data confirm the excellent general prognosis in CAC = 0 patients, quantify PET/CT results in a large population of these patients, define the risk for coronary ischemia, obstructive CAD, and other coronary pathologies requiring intervention, and document medium-term clinical outcomes.

## Figures and Tables

**Table 1:** Baseline Demographics of 4835 CAC = 0 Primary Prevention Non-ACS (Elective) Patients.

Variable	IB = 0%	IB >0–<10%	IB ≥ 10%	*p*-Value
N (%)	4479 (88.6%)	516 (10.2%)	59 (1.2%)	
Age	58.0 ± 12.3	58.7 ± 12.1	58.0 ± 14.2	0.4209
Female	3087 (68.9%)	358 (69.4%)	39 (66.1%)	0.8619
White	3979 (88.8%)	471 (91.3%)	56 (94.9%)	0.0943
BMI	31.3 ± 8.3	33.0 ± 8.6	33.0 ± 8.0	<0.0001
Family Hx	2198 (49.1%)	266 (51.6%)	34 (57.6%)	0.7529
Diabetes	820 (18.3%)	95 (18.4%)	11 (18.6%)	0.8991
Smoking	1225 (27.3%)	138 (26.7%)	15 (25.4%)	0.9206
HTN	2770 (61.8%)	333 (64.5%)	42 (71.2%)	0.1802
HLD	2497 (55.7%)	317 (61.4%)	38 (64.4%)	0.0213
ACEI ARB	434 (9.7%)	46 (8.9%)	13 (22.0%)	0.0127
OAC	526 (11.7%)	66 (12.8%)	14 (23.7%)	0.0251
Aspirin	671 (15.0%)	68 (13.2%)	16 (27.1%)	0.0244
BB	475 (10.6%)	72 (14.0%)	13 (30.5%)	<0.0001
CCB	151 (3.4%)	23 (5.4%)	8 (13.6%)	0.0002
Statin	446 (10.0%)	45 (8.7%)	13 (22.0%)	0.0118

Notes: ACEI = angiotensin converting enzyme inhibitor. ARB = angiotensin receptor blocker. BB = beta-blocker. BMI = body mass index; CCB = calcium channel blocker. HLD = hyperlipidemia (defined by Intermountain Health as total cholesterol ≥ 200 mg/dL or treatment with a lipid-lowering agent); HTN = hypertension; Hx = history; IB = ischemic burden; N = number; OAC = oral anticoagulant.

**Table 2:** Angiographic and Clinical Outcomes: Primary prevention Non-ACS (Elective) Patients.

Variable	IB = 0%	IB >0–<10%	IB ≥ 10%	*p*-Value
N (%)	4479 (88.6%)	516 (10.2%)	59 (1.2%)	
ICA ≤90 d	39 (0.9%)	56 (10.9%)	25 (42.4%)	<0.0001
ICA >90 d	91 (2.0%)	16 (3.1%)	8 (13.6%)	0.0104
Severe CAD ≤90 d	4 (0.1%)	4 (0.8%)	11 (18.6%)	<0.0001
Any revasc ≤90 d	2 (0.04%)	3 (0.6%)	10 (16.9%)	<0.0001
MACE ≤90 d	20 (0.4%)	3 (0.6%)	10 (16.9%)	<0.0001
--Pre-90 d Death	16 (0.4%)	0 (0%)	0 (0%)	0.5021
--Pre-90 d MI	3 (0.1%)	0 (0%)	0 (0%)	1.0000
--Pre-90 d CABG	0 (0%)	0 (0%)	6 (10.2%)	<0.0001
MACE ≤90 d	20 (0.4%)	3 (0.6%)	10 (16.9%)	<0.0001
--Pre-90 d PCI	2 (0.04%)	3 (0.6%)	5 (8.5%)	<0.0001
MACE >90 d	184 (4.1%)	26 (5%)	6 (10.2%)	0.0501
--Post-90 d Death	166 (3.7%)	20 (3.9%)	5 (8.5%)	0.1514
--Post-90 d MI	12 (0.3%)	4 (0.8%)	0 (0%)	0.1808
--Post-90 d CABG	2 (0.04%)	2 (0.4%)	0 (0%)	0.0988
--Post-90 d PCI	11 (0.2%)	0 (0%)	1 (1.7%)	0.1091
MACE total	202 (4.5%)	29 (5.6%)	16 (27.1%)	<0.0001
--Total Death	182 (4.1%)	20 (3.9%)	5 (8.5%)	0.2157
--Total MI	14 (0.3%)	4 (0.8%)	0 (0%)	0.2776
--Total CABG	2 (0.04%)	2 (0.4%)	6 (10.2%)	<0.0001
--Total PCI	12 (0.3%)	3 (0.6%)	6 (10.2%)	<0.0001

Notes CABG = coronary artery bypass grafting; CAD = coronary artery disease, with severe CAD defined as ≥70% diameter stenosis; d = days; IB = ischemic burden; MACE = major adverse cardiovascular event (death, non-fatal myocardial infarction, or coronary revascularization after 90 days); N = number; PCI = percutaneous coronary intervention; Revasc = revascularization; ICA = invasive coronary angiography.

## Data Availability

The data underlying this article cannot be shared publicly due to US privacy laws. The data are available upon reasonable request to the corresponding author.
